# Cardioprotective effect of crude polysaccharide fermented by *Trametes Sanguinea Lyoyd* on doxorubicin-induced myocardial injury mice

**DOI:** 10.1186/s40360-022-00641-y

**Published:** 2023-01-10

**Authors:** Chenjun Shen, Bo Yang, Lili Huang, Yueru Chen, Huajun Zhao, Zhihui Zhu

**Affiliations:** School of Pharmaceutical Sciences, Zhejiang Chinese Medical University, #548 Binwen Road, Hangzhou, 310053 China

**Keywords:** Doxorubicin, Cardiotoxicity, *Trametes Sanguinea Lyoyd* fermented crude polysaccharide, Cardioprotective effect

## Abstract

Doxorubicin (DOX) is a broad-spectrum anti-tumor drug, but its clinical application is greatly limited because of the cardiotoxicity. Thus, exploration of effective therapies against DOX-induced cardiotoxicity is necessary. The aim of this study is to investigate the effects and possible mechanisms of *Trametes Sanguinea Lyoyd* fermented crude polysaccharide (TSLFACP) against DOX-induced cardiotoxicity. We investigated the protective effects of TSLFACP on myocardial injury and its possible mechanisms using two in vitro cells of DOX-treated cardiomyocytes H9C2 and embryonic myocardial cell line CCC-HEH-2 and a in vivo mouse model of DOX-induced myocardial injury. We found that TSLFACP could reverse DOX-induced toxicity in H9C2 and CCC-HEH-2 cells. Similarly, we found that when pretreatment with TSLFACP (200 mg/kg, i.g.) daily for 6 days, DOX-induced myocardial damage was attenuated, including the decrease in serum myocardial injury index, and the amelioration in cardiac histopathological morphology. Additionally, immunohistochemistry and western blotting were used to identify the underlying and possible signal pathways. We found that TSLFACP attenuated the expression of LC3-II, Beclin-1 and PRAP induced by DOX. In conclusion, our results demonstrated that TSLFACP could protect against DOX-induced cardiotoxicity by inhibiting autophagy and apoptosis.

**Supplementary Information:**

The online version contains supplementary material available at 10.1186/s40360-022-00641-y.

## Introduction

Doxorubicin (DOX), an antibiotic, is known as one of the most widely used chemotherapeutic drugs and can be used to treat various cancers [1]. However, DOX produces some serious side effects after long-term use, especially cardiotoxicity, which is the most typical and serious adverse reaction [2]. DOX triggers irreversible myocardial dysfunction and heart failure [3]. Moreover, clinical studies have shown that DOX-induced cardiotoxicity is in a dose-dependent manner [4]. Therefore, the clinical application of DOX is greatly limited.

The mechanisms of DOX-induced myocardial injury were studied in recent years. DNA damage, apoptosis, oxidative stress, mitochondrial injury and autophagy were considered as the main factors of DOX-induced cardiotoxicity [5–8]. Among them, autophagy is a key process in DOX-induced myocardial damage. Briefly, DOX induces autophagic vesicles, and then results in the myocardial cell injury [9]. Besides, Cardiomyocyte apoptosis is well known to be a factor in cardiomyocyte injury [10]. Therefore, regulating autophagy and apoptosis is an effective preventive and therapeutic method against DOX-induced cardiotoxicity.

*Trametes Sanguinea Lyoyd* fermented crude polysacchade (TSLFACP) is a fermented crude polysaccharide isolated from *Trametes Sanguinea Lyoyd.* And increasing studies have demonstrated that *Trametes Sanguinea Lyoyd* polysaccharide has many bioactivities including antioxidant, antiviral, anti-inflammatory, antitumor and immune-enhancement activities [11, 12]. However, the effects of TSLFACP on DOX-induced cardiotoxicity remain largely unknown.

As such, the aim of the present study is to investigate the effect of TSLFACP on DOX-induced myocardial injury and the related mechanisms.

## Materials and methods

### Chemicals and materials

TSLFACP was isolated from Fermented Hemoglobin by professor Bo Yang from Zhejiang Chinese Medical University (Zhejiang, China). The fermentation and extraction process were as follows: the raw material was fermented for seven days at pH 5.5 and 150 r/min. The microbial fermentation broth was filtered to remove mycelia and insoluble components. The filtrate was concentrated to one fifth of the original volume under reduced pressure, and then added equal volume of ethyl acetate for extraction for three times. Then, the TSLFACP was obtained from the obtained aqueous phase which was prepared as described in the previous study [12].

Doxorubicin (DOX) was purchased from Hanhui Pharmaceuticals Co., Ltd. (Zhejiang, China). The assay kit for cardiactrofoninI (cTnI) was purchased from Meimian company (Jiangsu, China). The Cell Counting Kit-8 (CCK-8, cat. no. C0038), the hematoxylin and eosin (H&E) kit (cat. no. C0105S), DAPI staining solution (cat. no. C1002) and TUNEL apoptosis detection kit (cat. no. C1086) were purchased from Beyotime Biotechnology (Shanghai, China). Primary antibodies against SQSTM1/P62 (cat. no. #5114), LC3A (cat. no. #4599S), PARP (cat. no. #9532), Beclin-1 (cat. no. #3738S), caspase 3 (cat. no. #9662), GAPDH (cat. no. #2118) and β-actin (cat. no. #4970) were obtained from Cell Signaling Technology (Boston, USA). The secondary antibody of goat anti-rabbit IgM-HRP (cat. no. #7074) and enhanced chemiluminescence (ECL) reagent were obtained from BIO-RAD (Hercules, CA, USA).

### Mice and cell lines

All experiments in this study were conducted in accordance with the animal experiment guidelines of the Zhejiang Chinese Medical University Laboratory Animal Research Center (Approval No: IACUC-20181029-11). Male BALB/c mice weighing 25-28 g (Laboratory animal license number: SYXK (Zhejiang) 2019-0024; Laboratory animal quality certificate: SCXK (Shanghai) 2017-0005) were purchased from Slac laboratory animal Co., Ltd. (Shanghai, China). The mice were acclimatized at least one week prior to the experiment and housed under controlled environmental conditions of temperature (22 ± 2 ℃) in a 12 h light and dark cycle, and maintained on standard food pellets and tap water *ad libitum*.

Rat cardiomyocytes H9C2 cells were purchased from National Collection of Authenticated Cell Cultures (Shanghai, China). Human embryonic myocardial cell line CCC-HEH-2 were purchased from National Experimental Cell Resources Sharing Service Platform (Beijing Headquarters) (Beijing, China). All the cells were cultured in DMEM supplemented with 10% (v/v) fetal bovine serum (FBS), 100 U/mL penicillin and 100 μg/mL streptomycin at 37℃ under 5% CO_2_.

### Cell viability assay

Cell viability was detected by CCK-8 assay according to the manufacturer’s instructions as previously reported. Briefly, cells were cultured in a 96-well plate at the density of 3000 cells/well. After TSLFACP (25, 50, 100, 200 μg/mL) or DOX (2.5 μM) treatment, 10 μL CCK-8 reagent was added in each well for additional incubation at 37 ℃ for 2 h, and the optical density (OD) value was detected at 450 nm. Moreover, cell morphologies were imaged using an inverted microscope (100 ×).

### Western blotting analysis

Briefly, H9C2 cells were seeded (5 × 10^5^ per dish) in 6 cm^2^ dishes and pretreated with TSLFACP (200 μg/mL) for 12 h and then treated with DOX (2.5 μM) for another 12 h. Western blotting was performed using antibodies against SQSTM1/P62, LC3A, PARP, Beclin-1, caspase 3 and β-actin. SHST capture was used as an image acquisition tool, and AI 2020 was used as an image processing software package. Image J was used as protein quantitative analysis software.

### DOX-induced myocardial injury in vivo

DOX-induced myocardial injury in vivo was performed as described previously [13, 14]. Male BALB/c mice were randomly divided into three groups (*n* = 6): control group, DOX group and DOX + TSLFACP group. The mice of DOX group and DOX + TSLFACP group were subjected to a single intraperitoneal injection of 100 μL DOX (15 mg/kg), whereas those in the control group was injected with an equivalent volume of saline. The mice in the DOX + TSLFACP group were pretreated with 200 μL TSLFACP (200 mg/kg) for 6 consecutive days by intragastric administration prior to the injection of DOX, and administered for 6 consecutive days after the action of DOX. The daily weight of mice was recorded. Before the termination of the experiment, all mice were anesthetized by intraperitoneal injection of sodium pentobarbital (45 mg / kg), blood samples were obtained by tail-tip phlebotomy and then the hearts were taken for follow-up experiments by opening the thoracic cavity. The mice eventually died due to excessive blood loss in the whole process, and the mice did not feel severe pain. Serum samples were obtained by the centrifugation (3000 rpm, 4 ℃) for 10 min. Part of the heart tissue was fixed in formalin, and the rest were kept at -80 ℃.

### Measurement of cTnI level

For in vivo assay to determine the cardiac function, the serum samples of the mice were obtained. The level of cTnI in serum was detected by a commercial ELISA kit.

### Histopathologic assay

The heart tissue of mice was fixed in formalin, embedded in paraffin, and cut into slices with a thickness of 4 μm. The sections were then stained with H&E staining reagent. After that, the pathological changes were observed under a light microscope (100 ×).

### Immunohistochemistry assay

The heart tissue was fixed in formalin and embedded in paraffin, followed by being cut into 4-μm-thick slices. The slices were dewaxed and hydrated, and the endogenous peroxidase was blocked. Following the antigen repair, the section was sealed with 5% BSA and incubated with primary antibody at 4 ℃ overnight. HRP-labeled polymer was added on the second day, and the section was dyed with DAB, re-stained with hematoxylin, and sealed finally. After that, the pathological changes were observed under a microscope (100 ×).

### TUNEL staining

Cardiac slices were analyzed by TUNEL cell apoptosis assay kit according to the manufacturer’s instructions. Finally, the pathological changes were observed under a microscope (200 ×).

### Data analysis

All data were processed and analyzed by GraphPad 5.0. The measurement data were expressed by mean ± standard deviation $\left(\overline x\;\pm\;s\right)$. T test was used for comparison between two groups, and ANOVA was used for comparison among multiple groups. The difference was statistically significant with the threshold of *P* < 0.05.

## Results

### Analysis of polysaccharide content

**Fig. 1:**
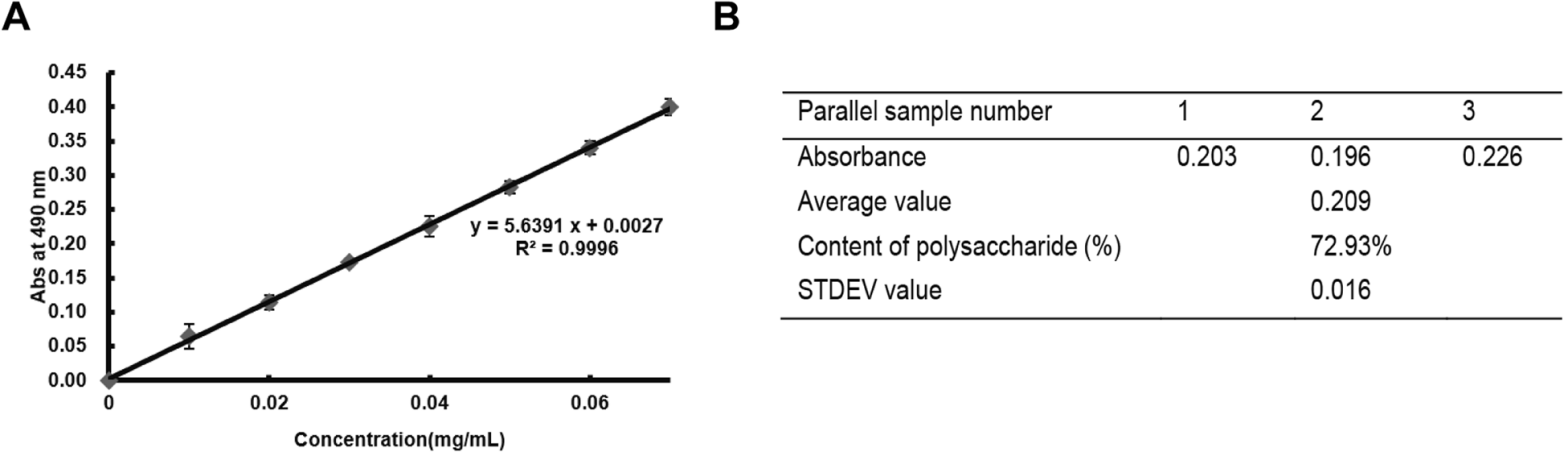
The content of TSLFACP. **A** Standard curve of glucose concentration. **B** Determination data of TSLFACP content (*n* = 3)

The content of TSLFACP was analyzed by phenol-concentrated sulfuric acid method. Glucose solutions of different concentrations were used to draw a standard curve. The curve showed that the polysaccharide concentration and absorbance in the range of 0 ~ 0.07 mg/mL had a great linear relationship (Fig. 1 A). Equal amounts of 0.05 mg/mL TSLFACP solutions were used for absorbance determination (*n* = 3), and the content of the polysaccharide was calculated to be 72.93% (Fig. 1 B) according to the standard curve.

### TSLFACP alleviates myocardial injury induced by DOX in vitro

**Fig. 2:**
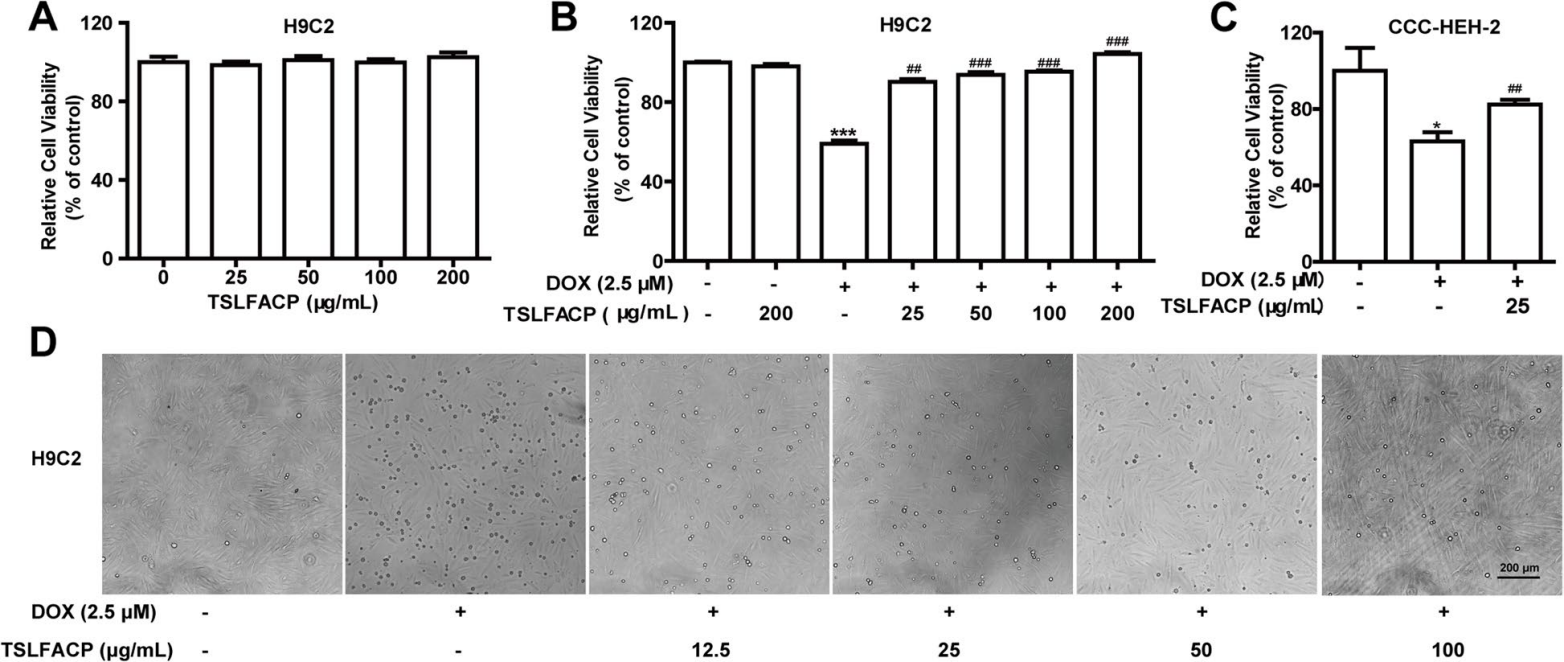
TSLFACP alleviates myocardial injury induced by DOX in vitro. **A** The effects of TSLFACP in different concentrations on the proliferation of H9C2 cells. **B** The effect of TSLFACP on the DOX-induced injury in the cardiomyocytes H9C2 cells. **C** The effect of TSLFACP on the DOX-induced injury in embryonic myocardial cell line CCC-HEH-2. **D** The morphology of H9C2 cells treated by DOX and TSLFACP (100 ×). The data is expressed as the *x* ± *s* (*n* = 4). *P < 0.05, ***P < 0.001 vs control; ^##^*P* < 0.01, ^###^*P* < 0.001 vs DOX

*Trametes Sanguinea Lyoyd* polysaccharide has many bioactivities including antioxidant, antiviral, anti-inflammatory, antitumor and immune-enhancement activities [11, 12]. In previous studies, we found that TSLFACP has cytoprotective effect on human umbilical vein endothelial cells (data is not shown). DOX has typical cardiotoxicity. Therefore, we hypothesized that TSLFACP could protect against DOX-induced injury of cardiomyocyte. Firstly, we examined the effects of TSLFACP on rat cardiomyocytes H9C2 cell proliferation by CCK-8 assay. We found that after treatment with different concentrations of TSLFACP (25, 50, 100, 200 μg/mL) for 24 h, the cell proliferation was no obvious changed (Fig. 2 A). Secondly, to assess the effect of TSLFACP on DOX-induced cardiotoxicity, we examined the effect of TSLFACP on DOX-induced rat cardiomyocytes H9C2 by CCK-8 assay. The cells were pretreated with different concentration of TSLFACP (25, 50, 100, 200 μg/mL) for 12 h and then treated with DOX (2.5 μM) for another 12 h. Compared with the control, the viability of H9C2 cells was significantly decreased after treatment with only 2.5 μM DOX and the viability of H9C2 cells was not change with only 200 μg/mL TSLFACP. But TSLFACP pretreatment (25, 50, 100, 200 μg/mL) significantly reversed the decrease of cell viability induced by DOX in a concentration-dependent manner (Fig. 2 B). Then, the effect of TSLFACP on DOX-induced human cardiomyocytes CCC-HEH-2 was examined by CCK-8 assay. TSLFACP could attenuate the inhibitory effects on DOX-induced cell viability (Fig. 2 C). Moreover, the numbers of H9C2 cells administrated with 2.5 μM DOX were evidently reduced and the remarkable changed morphology was observed, whereas the pretreated with different concentrations (12.5, 25, 50, 100 μg/mL) of TSLFACP significantly ameliorated the morphology of H9C2 cells (Fig. 2 D).

### TSLFACP alleviates myocardial injury induced by DOX in vivo

**Fig. 3:**
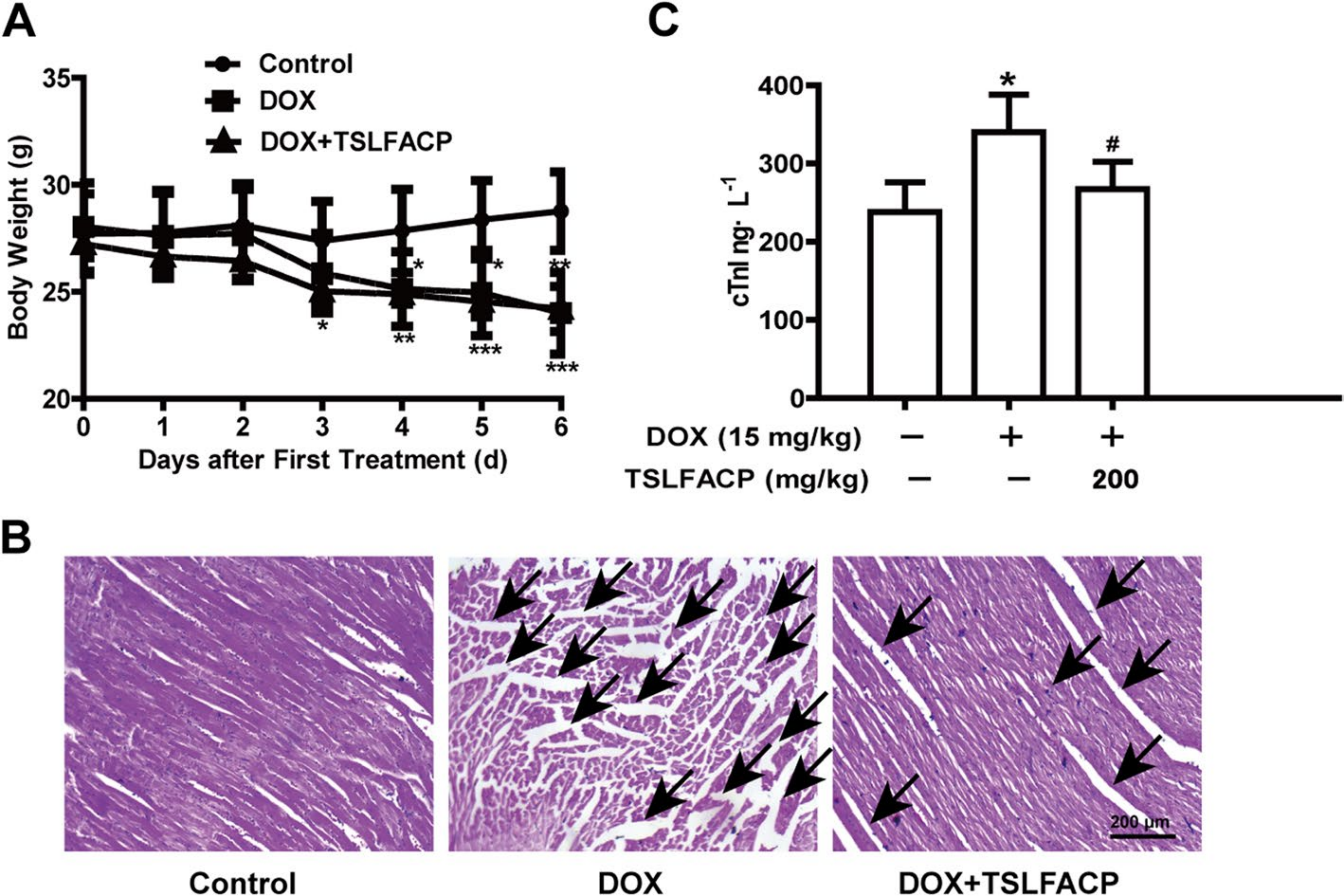
TSLFACP alleviates myocardial injury induced by DOX in vivo. **A** Changes in body weight of mice in the three groups. **B** Image of H&E staining on the heart tissue section of mice in these three groups (100 ×). **C** The level of cTnI in the serum of mice in each group. The data is expressed as the *x* ± *s* (*n* = 6). ^*^P < 0.05, *vs* control, ^#^*P* < 0.05 vs DOX

In addition, the effect of TSLFACP on DOX-induced cardiotoxicity in vivo was detected. We found that the body weight of mice in DOX group and DOX + TSLFACP group was decreased (Fig. 3 A). Following the collection of the heart and serum samples, H&E staining assay was performed for the histopathological examination on the heart tissues. We found that severe myocardium cells were damaged in the DOX group, characterized by myocardial fiber fracture, interstitial widening, local wavy cytoplasm dissolution and membrane rupture. When TSLFACP pretreatment, DOX-induced myocardial damage was alleviated (Fig. 3 B). The level of myocardial damage index cTnI in the serum was then evaluated. According to the results, DOX injection significantly elevated serums level of cTnI in mice, whilst TSLFACP pretreatment significantly reversed the elevated level of cTnI (Fig. 3 C, Table 1).

**Table 1:** Effect of TSLFACP on DOX-induced cTnI in vivo

	Ctrl	DOX (15 mg/kg)	DOX (15 mg/kg) + TSLFACP (200 mg/kg)
cTnI (ng/L)	238.19 ± 37.55	340.27 ± 47.81*	267.13 ± 35.03^#^

The date is expressed as the *x* ± *s* (*n* = 6). *P < 0.05 *vs* control; ^#^*P* < 0.05 *vs* DOX

### TSLFACP inhibits the DOX-induced autophagy in the cardiomyocyte

**Fig. 4:**
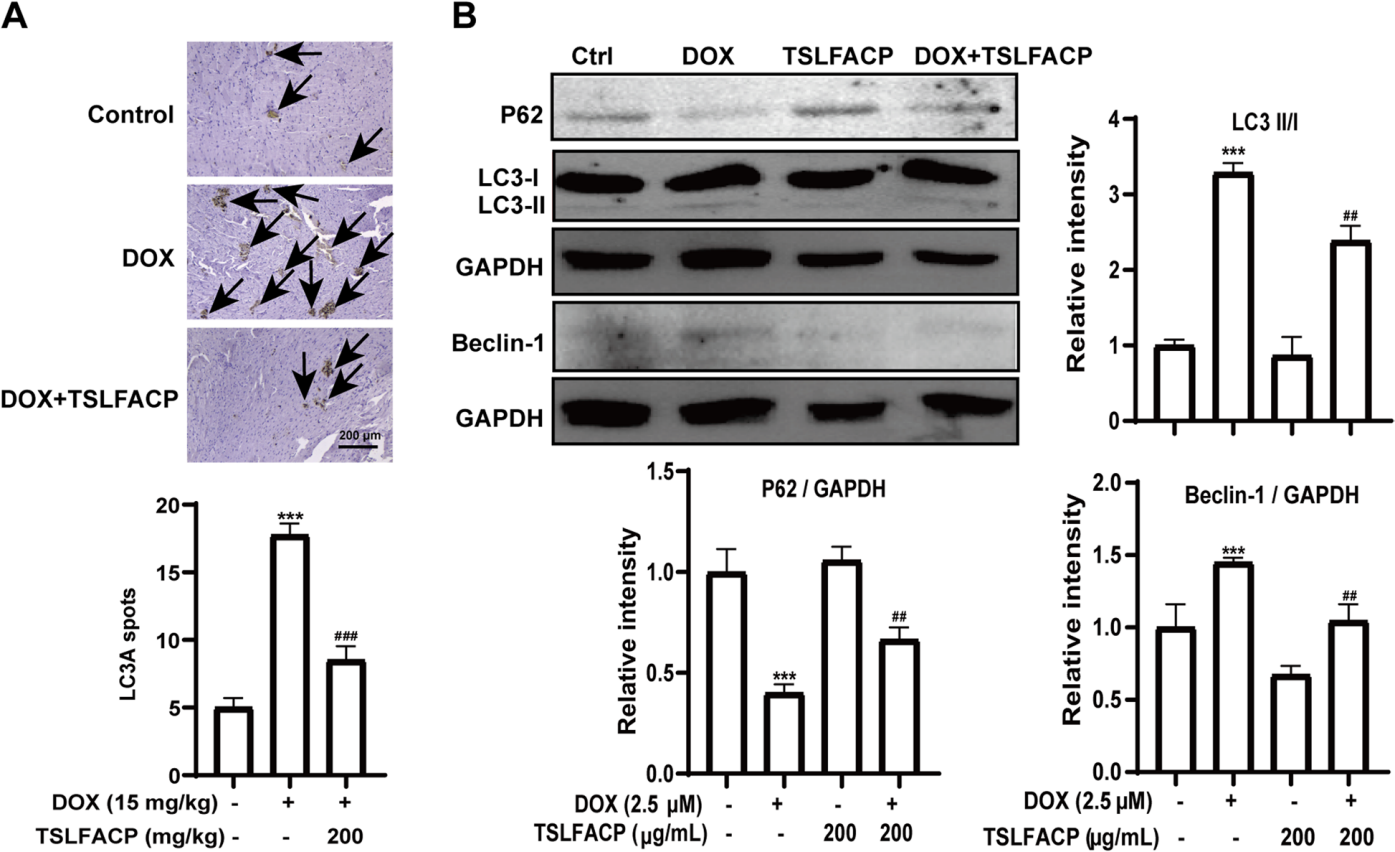
TSLFACP alleviates DOX-induced myocardial injury by reducing autophagy. **A** Intensity of LC3A spots expression in heart tissue samples (100 ×) and the number of LC3A spots was assessed. **B** Western blot analysis of autophagy-related proteins, the samples derive from the same experiment and that blots were processed in parallel, and blots shown were cut. ^*^P < 0.05, ^**^P < 0.01, ^***^P < 0.001 *vs* control, ^#^*P* < 0.05, ^###^*P* < 0.001 vs DOX

LC3A regulate several events leading to cellular and biochemical changes associated with autophagy and the appearance of LC3 spots means the accumulation of autophagy [14, 15]. Therefore, the activity of this typical autophagy-related protein was tested. The IHC assay was performed to investigate the effects of LC3A on the autophagy of cardiomyocyte. We found that the number of LC3A spots increased significantly in the heart tissue treated with DOX, and TSLFACP preconditioning reversed the effects of DOX by increasing the protein expression of LC3A (Fig. 4 A). To further explore the mechanism by which TSLFACP alleviates DOX-induced myocardial autophagy, H9C2 cells were used. LC3-I is lipidated to LC3-II, which binds LC3 to autophagic vesicles, a hallmark of autophagy. And Beclin-1 stimulates autophagy [16]. P62 binds to the autophagosome membrane protein LC3/Atg8, thereby transporting protein aggregates containing P62 to the autophagosome, and the degradation of the autophagosome by lysosomes leads to a decrease in the level of P62 [17]. In our result, DOX reduced the expression of p62, increased the expression of Beclin-1 and induced lipidation of LC3-I into LC3-II. However, TSLFACP ameliorated the effect of DOX on these proteins (Fig. 4 B).

### TSLFACP inhibits the DOX-induced apoptosis in the cardiomyocyte

**Fig. 5:**
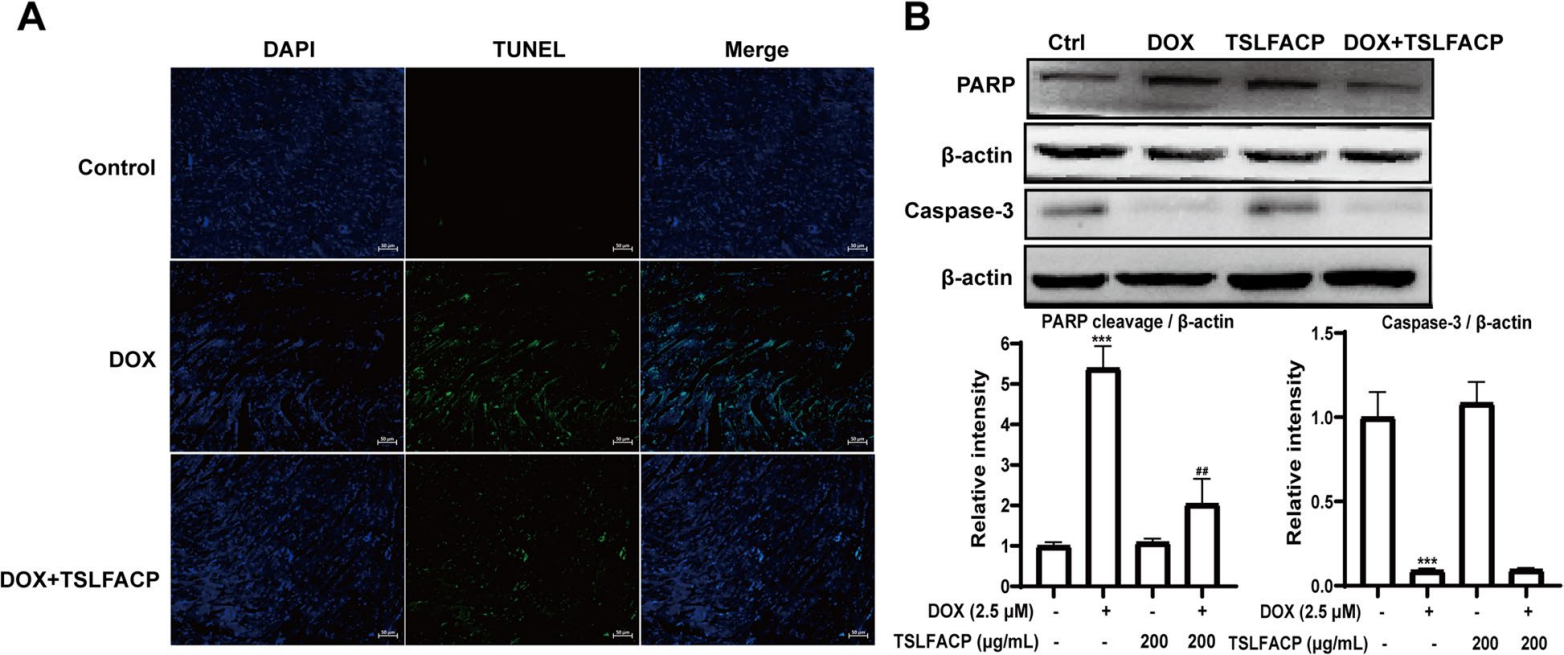
TSLFACP alleviates DOX-induced myocardial injury by reducing apoptosis. **A** Tunel staining in heart tissue samples (200 ×). **B** Western blot analysis of apoptosis-related proteins, the samples derive from the same experiment and that blots were processed in parallel, and blots shown were cut. ^***^P < 0.001 *vs* control, ^##^*P* < 0.01, ^###^*P* < 0.001 vs DOX

Besides autophagy, apoptosis is also considered to be an important cause of myocardial injury [18]. The effect of DOX and TSLFACP on apoptosis was detected by tunel staining. We found that compared with the control group, the green fluorescence in the DOX group was stronger; while TSLFACP pretreatment could significantly attenuate the appearance of green fluorescence (Fig. 5 A), indicating that TSLFACP attenuated DOX-induced apoptosis. To further explore how the TSLFACP formula alleviates DOX-induced apoptosis, H9C2 was used. After treatment with DOX, the cleaved PRAP was appeared, and the expression of caspase-3 was reduced. However, TSLFACP pretreatment reduced the appearance of cleaved PRAP (Fig. 5 B).

## Discussion

DOX is commonly used for the treatments of solid and hematological malignancies. However, it produces many side effects, especially cardiotoxicity. Clinically, the DOX-induced cardiotoxicity is irreversible and dose-dependent, which leads to the poor prognosis of patients [19]. DOX-induced cardiomyopathy is characterized by progressive heart failure, which causes progressive myocardial cell death [20]. The mortality rate of patients will significantly increase once heart failure occurs [21]. Therefore, the cardiotoxicity induced by DOX limits its clinical application. Hence, it is particularly important to prevent or alleviate DOX-induced cardiotoxicity, which has become a research hotspot in recent years.

*Trametes Sanguinea Lyoyd* is a kind of fungi with homology of medicine and food, which has important value. There are many bioactive substances in *Trametes Sanguinea Lyoyd*, among which polysaccharide has been proved to be the most important bioactive substance [12]. Polysaccharide, as a bioactive substance in most Traditional Chinese Medicines, has been proved to have protective effects on heart, liver, kidney and other organs [22–24]. Some scholars have found that Ganoderma lucidum polysaccharide can alleviate the adverse effects of DOX on myocardial cells [25]. It has been demonstrated that astragalus polysaccharide can restore DOX-induced autophagy disorder and inhibit DOX-induced cardiomyocyte apoptosis as well [26]. These findings suggest that TSLFACP, which is isolated from fermented *Trametes Sanguinea Lyoyd*, may have a cardiac protective effect as well.

In the current study, we investigated the effect of TSLFACP on DOX-induced cardiotoxicity. The enhancement on the survival of cardiomyocytes is proposed to be pivotal to blunting the deteriorated structure and function of myocardium following the damage [27]. Due to the strong cytotoxicity of DOX, we detect the effect of TSLFACP on cell proliferation of rat cardiomyocytes H9C2 firstly. Unsurprisingly, the TSLFACP was proved with no toxicity to the cardiomyocytes H9C2. Then, we found that TSLFACP pretreatment greatly ameliorated the death and cellular damage of DOX-induced H9C2 and CCC-HEH-2 cardiomyocytes in vitro. This discovery provided confidence for us to study the effect of TSLFACP on DOX-induced cardiotoxicity in vivo. The toxicity of DOX to the heart often causes weight loss and pathological changes in the heart tissue, such as unclear texture of the heart tissue, infiltration of inflammatory cells, as well as affects the levels of lactate dehydrogenase (LDH), MB (CK-MB), cTnI and other myocardial injury indexes [28]. cTnI is a regulatory protein of myocardial muscle, and it can be rapidly introduced into the blood from cells when myocardial cells are injured [29]. Additionally, its slow degradation and the long diagnosis time window make it become a commonly used index for detecting myocardial injury in clinic. In our study, we found pretreatment with TSLFACP ameliorated myocardial structure damage and decreased the level of cTnI in vivo. Regrettably, TSLFACP did not alleviate DOX-induced weight loss.

Autophagy is a phagocytosis process of cytoplasmic proteins or organelles into vesicles and fusion with lysosomes to form autophagic lysosomes and degrade their encapsulated contents [30]. Autophagy can occur in both physiological and pathological processes of the body. Simply put, the degraded substance binds to the autophagy substrate SQSTM1/P62 when autophagy occurs, which is concurrent with the binding of the lipidized LC3 (LC3-II) to the autophagy vesicles [31]. The autophagy, therefore, is activated. And Beclin-1 promotes the localization of autophagic proteins to autophagic vacuoles. In the heart, dysregulated autophagy induces death of cardiomyocytes and actively mediates cardiac injury and dysfunction in some conditions, including reperfusion injury, doxorubicin cardiomyopathy, and lysosomal storage disorders [32]. Existing investigation has documented the efficacy of polysaccharide, *Lycium barbarum* polysaccharide to be exact, on inhibiting autophagy so as to protect the cardiomyocytes [33]. This discovery made us wondered if TSLFACP could inhibit autophagy. And accordingly, we detected the protein levels of LC3A in mice heart tissue sections by immunohistochemistry. It became clearly that there were many LC3A-positive spots in the heart sections after DOX treatment, which demonstrates the activation of autophagy. However, LC3A-positive spots of heart tissue were reduced after TSLFACP administration, which means autophagy is relieved. Subsequently, H9C2 cells were used to further explore the mechanism by which TSLFACP alleviates DOX-induced myocardial autophagy in vitro. Autophagy was activated after DOX treatment of H9C2, manifested in decreased p62, increased Beclin-1, and lipidation of LC3-I into LC3-II. However, TSLFACP ameliorated the effect of DOX on these proteins, which suggests that TSLFACP alleviate DOX-induced autophagy.

Besides autophagy, DOX-induced cardiac injury is mostly identified by apoptosis [21]. When apoptosis is initiated, genomic DNA breaks, and the exposed 3'-OH is labeled with FITC, which can be observed by fluorescence microscopy. A polysaccharide of Dendrobium officinale play a cardioprotective role by alleviating H_2_O_2_-induced apoptosis in H9C2 cardiomyocytes [10]. And a novel honeysuckle polysaccharide inhibits apoptosis of mice cardiomyocytes [34]. These studies suggest that TSLFACP may inhibit DOX-induced cardiomyocyte apoptosis. Therefore, cardiac slices were used to detect cardiomyocyte apoptosis using TUNEL kit. Excitingly, TSLFACP attenuated DOX-induced apoptosis. Besides, the cleaved PRAP was appeared, and the expression of caspase-3 was reduced after DOX treatment. However, TSLFACP pretreatment reduced the appearance of cleaved PRAP. The 116kD PARP is cleaved into 31kD and 85kD fragments by caspase-3 when apoptosis occurs, so that PARP cannot function normally. PARP assists cells in maintaining viability, and PARP cleavage promotes cell disintegration and serve as a marker of apoptosis [35]. These imply that TSLFACP alleviate DOX-induced apoptosis.

## Conclusions

In summary, this is the first study to show that the TSLFACP exerts a cardioprotective effect in a DOX-induced mice model. Specifically, TSLFACP inhibits the autophagy of cardiomyocyte via the inhibition of LC3A-related autophagy signaling pathway and inhibits the apoptosis of cardiomyocyte via the inhibition of PARP-related apoptosis signaling pathway. Overall, we believe that TSLFACP should be a promising polysaccharide to prevent DOX-induced myocardial injury in the future.

## Supplementary Information

**Additional file 1.**

## Data Availability

The datasets used and/or analyzed during the current study available from the corresponding author on reasonable request.
