# Correction: Cardioprotective effect of crude polysaccharide fermented by Trametes Sanguinea Lyoyd on doxorubicin-induced myocardial injury mice

**DOI:** 10.1186/s40360-023-00659-w

**Published:** 2023-03-20

**Authors:** Chenjun Shen, Bo Yang, Lili Huang, Yueru Chen, Huajun Zhao, Zhihui Zhu

**Affiliations:** School of Pharmaceutical Sciences, Zhejiang Chinese Medical University, #548 Binwen Road, Hangzhou, 310053 China

**Correction: BMC Pharmacol Toxicol 24, 1 (2023)**

**https://doi.org/10.1186/s40360-022-00641-y**

Following publication of the original article [1], the authors identified an error in the author name of Huajun Zhao. The given name and family name were erroneously transposed.

The incorrect author name is: Zhao Huajun

The correct author name is: Huajun Zhao

The author group has been updated above and the original article [1] has been corrected.
