# Supplementary figures and images for: Cardioprotective effect of crude polysaccharide fermented by Trametes Sanguinea Lyoyd on doxorubicin-induced myocardial injury mice
Source: BMC Pharmacol Toxicol. 2023 Jan 10;24:1. doi: 10.1186/s40360-022-00641-y (PMC9832647; doi:10.1186/s40360-022-00641-y)

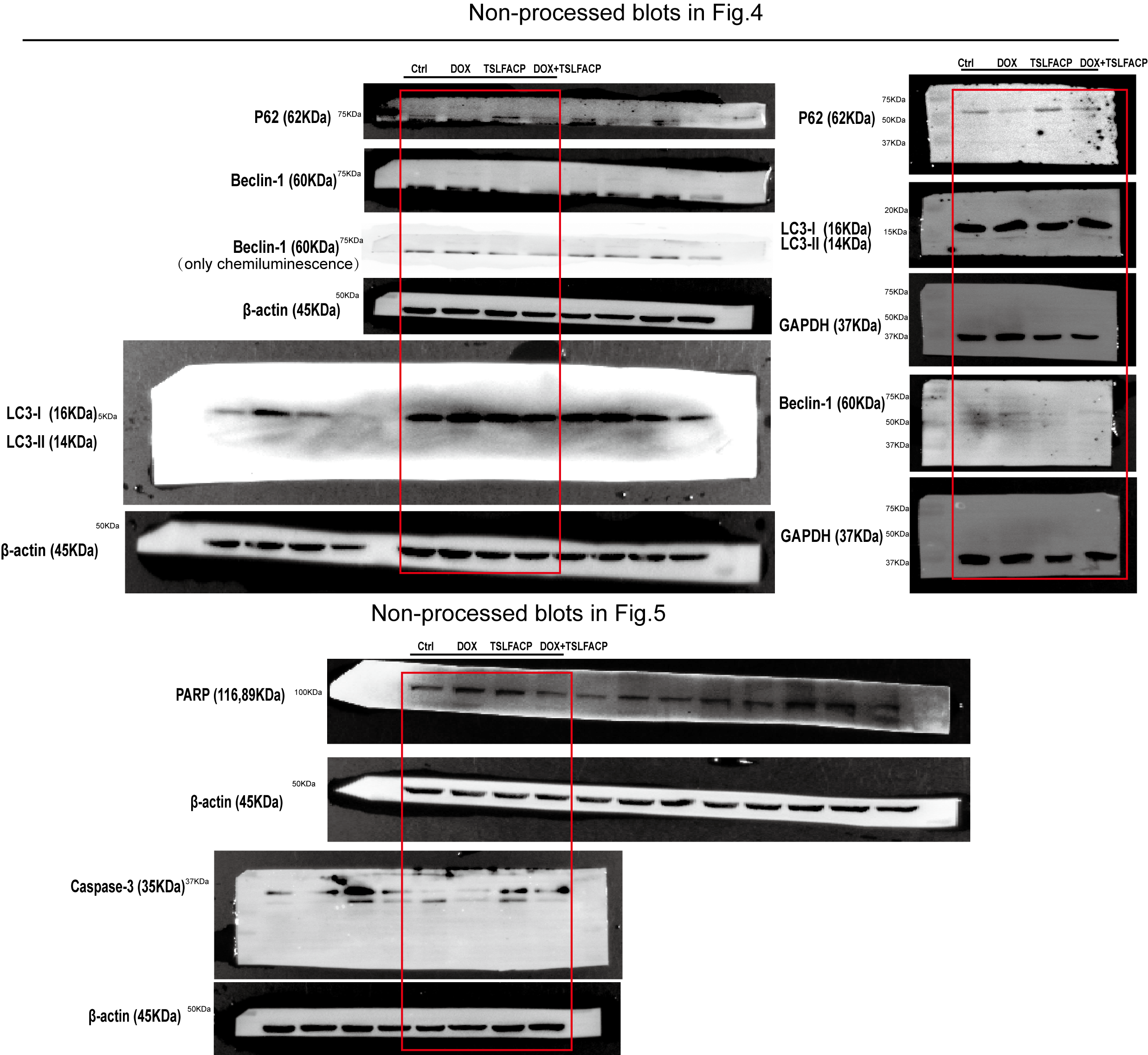

Supplement: Supplementary file 1 — Additional file 1. [file 40360_2022_641_MOESM1_ESM.tif]
